# Effects of local authority expenditure on childhood obesity

**DOI:** 10.1093/eurpub/cky252

**Published:** 2018-12-07

**Authors:** Dan Liu, Anne Mason, Linda Marks, Howard Davis, David J Hunter, Llinos Mary Jehu, Joanne Smithson, Shelina Visram

**Affiliations:** 1 Centre for Health Economics, University of York, York, UK; 2 Centre for Public Policy and Health, School of Medicine, Pharmacy and Health, Durham University Queen’s Campus, Stockton-on-Tees, UK; 3 Centre for Communities and Social Justice, Coventry University, Coventry, UK; 4 Institute of Health & Society, Faculty of Medical Sciences, Newcastle University, Newcastle upon Tyne, UK; 5 School of Social Policy, University of Birmingham, Edgbaston, Birmingham, UK; 6 Voluntary Organisations' Network North East, MEA House, Ellison Place, Newcastle upon Tyne, UK

## Abstract

**Background:**

Under the 2013 reforms introduced by the Health and Social Care Act (2012), public health responsibilities in England were transferred from the National Health Service to local authorities (LAs). Ring-fenced grants were introduced to support the new responsibilities. The aim of our study was to test whether the level of expenditure in 2013/14 affected the prevalence of childhood obesity in 2016/17.

**Methods:**

We used National Child Measurement Programme definitions of childhood obesity and datasets. We used LA revenue returns data to derive three measures of per capita expenditure: childhood obesity (<19); physical activity (<19) and the Children’s 5–19 Public Health Programme. We ran separate negative binomial models for two age groups of children (4–5 year olds; 10–11 year olds) and conducted sensitivity analyses.

**Results:**

With few exceptions, the level of spend in 2013/14 was not significantly associated with the level of childhood obesity in 2016/17. We identified some positive associations between spend on physical activity and the Children’s Public Health Programme at baseline (2013/14) and the level of childhood obesity in children aged 4–5 in 2016/17, but the effect was not evident in children aged 10–11. In both age groups, LA levels of childhood obesity in 2016/17 were significantly and positively associated with obesity levels in 2013/14. As these four cohorts comprise entirely different pupils, this underlines the importance of local drivers of childhood obesity.

**Conclusions:**

Higher levels of local expenditure are unlikely to be effective in reducing childhood obesity in the short term.

## Introduction

Childhood obesity is reaching epidemic proportions, affecting low- and middle-income countries as well as higher income countries.^1^ The impacts of childhood obesity include physical and mental health effects, educational attainment and quality of life. Children with obesity are at higher risk of being obese in adulthood and are at increased risk of developing cardiovascular disease, Type 2 diabetes and hypertension in later life.^2^

In 2016/17, almost one quarter of English children aged 4–5 were overweight or obese and in those aged 10–11 the corresponding figure was one-third.^3^ In 2014/15, the National Health Service (NHS) in England spent £5.1 billion on overweight and obesity-related ill-health,^2^ whilst local government expenditure on obesity totalled £36.6 million for children and £63.1 million for adults.^4^

In April 2013, the Health and Social Care Act 2012 transferred Public Health responsibilities in England from the NHS to upper tier and single tier local authorities (LAs) along with a ring-fenced public health budget.^5^ In England, local government comprises lower tier, upper tier and single tier LAs. Some areas have both district councils (lower tier) and county councils (upper tier). Single tier means London borough councils, metropolitan district councils and unitary authorities.

Public health budgets are held by upper tier and single tier LAs, which are required to report annually on how they spent their budgets by pre-defined categories. Some types of spend are mandatory, others are discretionary. During the first three years of the April 2013 reforms, there were 18 public health budget reporting categories, three of which are particularly relevant for their potential impact on childhood obesity: obesity in children aged 0–19; physical activity in children aged 0–19; and the Children’s 5–19 Public Health Programmes.

1. Obesity – children: includes expenditure on items such as ‘proactive follow-up’ to the mandatory National Child Measurement Programme (NCMP) (described below); weight management interventions for children and obesity prevention programmes.
2. Physical activity – children: includes active travel initiatives, sports-based interventions and community-based schemes targeted at children.
3. Children 5–19 Public Health Programmes: e.g. Healthy Schools Programme, school nursing services, Healthy Child Programme and health promotion/prevention interventions.^6^

As none of these categories is mandatory for LAs, they have discretion over their levels of spend. It is plausible that LAs with higher levels of childhood obesity may have spent more in tackling this issue and that the subsequent impact on childhood obesity levels would be greater than in authorities with less capacity to improve. Data on the services funded are not routinely collected, but the level of per capita spend can be seen as indicative of the importance that LAs place on tackling the problem of childhood obesity relative to the other competing demands on their public health budgets.

This study aimed to test the effects of these three types of LA expenditure in 2013/14 on childhood obesity in 2016/17, three years after the reforms. This study contributes to the literature on the relationship between local expenditure and childhood obesity as there is limited evidence on this topic.

## Methods

We used panel data with LAs as the unit of analysis. There are 152 LAs in England, but some datasets report combined values for Cornwall and Isles of Scilly, and for Hackney and the City of London. Therefore, our dataset included 150 upper tier and single tier LAs.

### Outcome measures

Introduced in 2005/6, the NCMP collects height and weight measurement of over one million children each year in Reception (aged 4–5 years old) and in Year 6 (aged 10–11 years old) in state-maintained schools in England.^7^ The programme is a prescribed (mandatory) function for LAs and has been funded under a bespoke category of the public health budget since the 2013 reforms.

**Figure 1:**
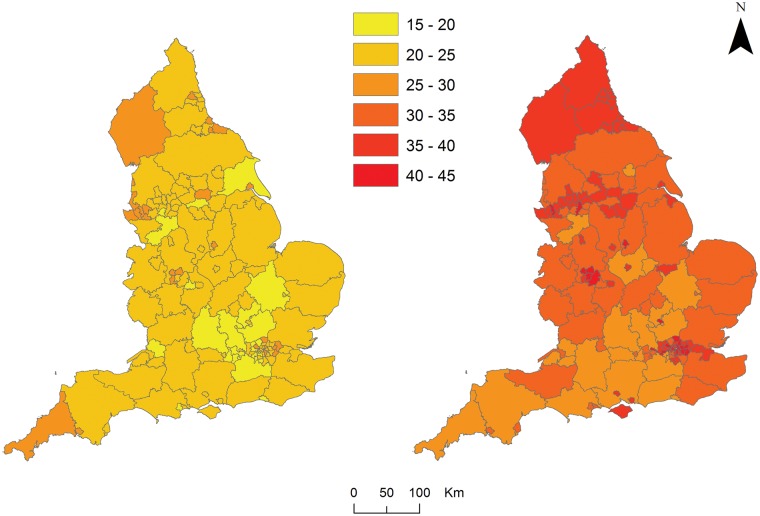
The prevalence of overweight and obesity for children aged 4–5 (left) and aged 10–11 (right) at the upper tier and single tier LA level in England (2016/17)

The NCMP classifies children as overweight or obese if their body mass index is on or above the 85th centile of the British 1990 growth reference according to age and sex.^8^ Following the definition of obesity adopted by the NCMP, the outcome variable in our study is the proportion of children in each of the school years who were overweight or obese in 2016/17. The geographical variation in patterns of childhood obesity is shown in figure 1 (Colour figure is available as Supplementary data online.).

### Main explanatory variables—measures of spend

We calculated 2013/14 LA per capita actual net current expenditure on (i) childhood obesity, (ii) physical activity (for children) and (iii) Children 5–19 Public Health Programme, by dividing total spend in each category by the relevant LA population aged 5–19 years old. The spend data were sourced from LA revenue returns data, and the population data were published by the Office for National Statistics (ONS). For the baseline model, we took terciles of per capita expenditure for 2013/14, i.e. low, medium and high categories as the principal explanatory measures.

### Covariates

We reviewed studies published from 2010 to 2016 on non-interventional factors predicting childhood obesity to inform the selection of control variables. Over 3500 potentially relevant records were screened, 70 papers were assessed for eligibility and 53 were found to be relevant. Six were from the UK, seven from Australia, 23 from the USA and two were set in multiple countries. The remaining studies were from Canada, Denmark, Finland, Germany, the Netherlands, Norway and Sweden.

Based on the findings of the literature review and data availability, we generated the following covariates to control for LA factors: gender [the percentage of males in each age group (4–5 and 10–11 year olds)]; rurality (% rural including market towns);^9^^,^^10^ deprivation {% population living in 20% most deprived small areas [lower layer super output areas (LSOAs)]}; ethnicity (% pupils who are not white British in primary school);^9–26^ access to fast-food outlets (outlets per 100 000 population)^14^ and type of LA, based on categories used for national reporting activity. The four types comprised: shire counties; unitary authorities; metropolitan districts and London boroughs. Unless otherwise stated, all covariates were derived from 2016/17 data. We also included the three-year-lagged prevalence of LA childhood obesity in 2013/14.^18^^,^^27^^,^^28^ The data on rurality (2011) and deprivation (2015) came from the ONS. The measure of ethnicity was sourced from the Department for Education’s National Statistics, and Public Health England publishes data on local density of fast-food outlets.

### Modelling

When assessing causation between a factor and outcome, a necessary condition is that the cause precedes the outcome.^29^ As the NCMP collected data on children’s weight throughout a school year, the sequencing of concurrent expenditure and outcomes cannot be determined from routine data. In addition, expenditure on interventions to reduce childhood obesity is unlikely to have an immediate effect. Therefore, we estimated the effect of the lagged values (2013/14) of spend on childhood obesity in 2016/17 (the latest year available).

We used random effects negative binomial models to deal with overdispersion in the outcome variables. We ran two models to test the effects of spend on the proportion of children aged 4–5 who were overweight or obese and on those aged 10–11.

### Sensitivity analyses

To test the robustness of our findings we undertook three sensitivity analyses. In the base case model, spend was measured as terciles of per capita expenditure. In the sensitivity analyses, the outcome variable was unchanged but we tested different measures of spend:

1. Per capita spend in 2013/14 (a continuous measure);
2. Mean per capita expenditure across 2013/14 and 2014/15 (in terciles);
3. The sum of expenditure on childhood obesity, physical activity and the children’s Public Health programme as a percentage of the total public health spend in 2013/14.

## Results

Summary statistics of the estimation sample are shown in table 1. In 2013/14, LA spend on childhood obesity averaged £3.84 per child (5–19), equivalent to 1.1% of the total spend on public health. The corresponding figure for children’s physical activity was £2.50 (0.8%) and for the Children’s 5–19 Public Health Programme was £28.41 (9.7%). There was considerable variation in these statistics across LAs.

**Table 1:** Summary statistics: estimation sample (*N*=150)

	Variables	Mean	SD
Outcomes	Children aged 4–5 overweight or obese	949	706
	Children aged 4–5 (all)	4196	3108
	Children aged 10–11 overweight or obese	1270	894
	Children aged 10–11 (all)	3710	2751
Expenditure—per capita (£), 2013/14	Obesity (children) (A)	3.84	6.42
	Physical activity (children) (B)	2.5	6.7
	Children Public Health Programme (C)	28.41	17.28
Expenditure—other, 2013/14	Spend on A+B+C as % total public health spend	11.69	4.87
Controls—lagged outcomes	% children aged 4–5 overweight or obese, 2013/14	22.76	2.45
	% children aged 10–11 overweight or obese, 2013/14	34.09	3.86
Controls—other	% males aged 4–5	51.17	0.79
	% males aged 10–11	51.18	0.83
	% population living in rural areas, 2011	17.51	24.55
	% living in 20% most deprived LSOAs, 2015	24.85	19.02
	% minority ethnicity	34.08	25.89
	Fast-food outlets per 100 000 population	90.48	24.96
Controls—LA class	London boroughs	0.21	0.41
	Metropolitan districts	0.24	0.43
	Unitary authorities	0.37	0.48
	Shire counties	0.18	0.39

All variables are for 2016/17, unless stated otherwise. SD: standard deviation.

In the base case analysis, for children aged 4–5, LAs with medium levels of per capita spend in 2013/14 on either childhood obesity or on the children’s public health programme had significantly higher levels of childhood obesity in 2016/17 compared to authorities with low levels of spend (table 2). None of the other measures of LA spend in 2013/14 was significantly associated with the proportion of obese or overweight children in 2016/17 in either age group.

**Table 2:** Results from the base case regression analyses of childhood obesity (*N*=150)

		Aged 4–5		Aged 10–11	
		IRR^a^	95% CI^b^	IRR	95% CI
Per capita spend (terciles, 2013/14)	Obesity: medium	0.994	[0.961, 1.028]	0.997	[0.978, 1.017]
	Obesity: high	0.996	[0.965, 1.027]	1.004	[0.982, 1.026]
	Physical activity: medium	1.033*	[1.003, 1.065]	0.991	[0.970, 1.012]
	Physical activity: high	1.007	[0.970, 1.045]	1.001	[0.979, 1.022]
	Public Health Programme: medium	1.040*	[1.008, 1.074]	1.011	[0.992, 1.030]
	Public Health Programme: high	1.011	[0.977, 1.047]	1.006	[0.984, 1.030]
Controls—lagged outcomes	% children aged 4–5 overweight or obese, 2013/14	1.031^***^	[1.024, 1.038]		
	% children aged 10–11 overweight or obese, 2013/14			1.026^***^	[1.022, 1.030]
Controls—other	% males aged 4–5	0.995	[0.977, 1.013]		
	% males aged 10–11			1.007	[0.998, 1.017]
	% population living in rural areas, 2011	1	[0.999, 1.001]	1	[0.999, 1.001]
	% living in 20% most deprived LSOAs, 2015	1.001^**^	[1.000, 1.002]	1.001	[1.000, 1.002]
	% minority ethnicity	1	[0.999, 1.001]	1	[1.000, 1.001]
	Fast-food outlets per 100 000 population	1	[1.000, 1.001]	1	[0.999, 1.000]
Controls—LA class	London boroughs	0.958*	[0.922, 0.996]	0.989	[0.948, 1.032]
	Metropolitan districts	0.986	[0.951, 1.023]	1.005	[0.983, 1.027]
	Shire counties	1.007	[0.970, 1.046]	0.989	[0.964, 1.014]

Exponentiated coefficients from negative binomial model.

* *P *< 0.05.

** *P *< 0.01.

*** *P *< 0.001.

All variables are for 2016/17, unless stated otherwise.

a IRR: Incidence rate ratios (values above 1 indicate a positive effect, values below 1 show a negative effect).

b CI: Confidence interval.

In both age groups, a 1% point increase in LA levels of childhood obesity in 2013/14 was associated with an increase in childhood obesity in 2016/17 of 3.1% for children aged 4–5 years old and of 2.6% for children aged 10–11 years old. As these four cohorts comprise entirely different pupils, this provided evidence of geographical drivers of childhood obesity. In the younger age group only, higher levels of LA deprivation were associated with higher levels of obesity, and London boroughs had significantly lower levels of obesity compared to unitary authorities (the reference group).

The sensitivity analyses tested whether the relationship between spend and outcomes depended on how LA spend was measured (table 3). When per capita spend was entered in the model as a continuous variable, spend on children’s physical activity (in 2013/14) was positively related to childhood obesity but only in 10–11 year olds (in 2016/17). No other coefficient was statistically significant. In the model that captured the mean per capita expenditure across 2013/14 and 2014/15 (in terciles), LAs with medium levels of spend on physical activity in 2013/14 had significantly higher levels of childhood obesity in children aged 4–5 in 2016/17 compared to lower spending authorities. None of the other measures of spend was significantly related to levels of childhood obesity.

**Table 3:** Results from the sensitivity analyses

	Aged 4–5		Aged 10–11	
	IRR^a^	95% CI^b^	IRR	95% CI
Analysis 1:				
Per capita spend [2013/14]				
Childhood obesity	1.000	[0.998, 1.002]	0.999	[0.998, 1.000]
Children's physical activity	1.000	[0.999, 1.001]	1.001*	[1.000, 1.002]
Children Public Health Programme	1.000	[0.999, 1.001]	1.000	[1.000, 1.001]
Analysis 2:				
Terciles of mean per capita spend [2013/14 to 2014/15]				
Childhood obesity: medium	1.002	[0.974, 1.030]	0.994	[0.976, 1.012]
Childhood obesity: high	0.990	[0.963, 1.019]	0.989	[0.968, 1.009]
Children's physical activity: medium	1.028*	[1.001, 1.056]	0.996	[0.978, 1.014]
Children's physical activity: high	1.024	[0.992, 1.057]	1.001	[0.981, 1.021]
Children Public Health Programme: medium	1.020	[0.991, 1.050]	1.007	[0.988, 1.026]
Children Public Health Programme: high	1.021	[0.990, 1.053]	1.003	[0.980, 1.027]
Analysis 3:				
% total public health spend [2013/14]				
(Childhood obesity + Children's physical activity + Children Public Health Programme) / Total public health spend	1.001	[0.998, 1.003]	1.001	[1.000, 1.003]

Exponentiated coefficients from negative binomial model.

* *P *< 0.05.

All variables are for 2016/17, unless stated otherwise.

a IRR: Incidence rate ratios (values above 1 indicate a positive effect, values below 1 show a negative effect).

b CI: Confidence interval.

## Discussion

In 2013, responsibilities and budgets for public health in England were transferred from the NHS to upper tier and single tier LAs. Our study investigated whether the amount spent per head in 2013 on childhood obesity, physical activity for children, and the Children’s Public Health Programme had an impact on childhood obesity three years later. We found no evidence that higher levels of expenditure were associated with lower levels of obesity in children aged 4–5 or in those aged 10–11. Most analyses found no relationship between spend and childhood obesity, although we identified a small number of positive associations between spend on physical activity and Children’s Public Health Programme at baseline (2013/14) and the level of childhood obesity in children aged 4–5 in 2016/17. This finding could be a phenomenon known as ‘reverse causality’: if childhood obesity is a persistent and intractable local problem, as our findings suggest, higher spend by LAs at baseline could represent a recognition of and response to this problem.

This study contributes new evidence in several respects. This is the first study to look at the effects of the public health reforms by examining the link between LA spend at baseline and LA levels of childhood obesity three years later. In addition, this is a national study that used data on LAs in England except for two small authorities (for whom data are not consistently reported). Lastly, to control for confounding effects on childhood obesity, we included a range of non-interventional factors in our models, which we identified from an evidence review.

A key limitation of our study is there were just three years of follow-up since the April 2013 reforms. The effects of LA spend are unlikely to occur in the short term, and our study cannot rule out the possibility of longer-term impacts of spend on childhood obesity. Provided NCMP data continue to be reported annually, future research could test for possible longer-term effects of spend on outcome. In the meantime, robust inferences regarding the nature of the relationship between spend and outcomes are unwarranted. Another limitation is that our analysis was based on data from children aged 4–5 and 10–11 as these are the only age groups assessed in the NCMP.

Our analyses relied on LA level data. Individual level data would have enabled a multilevel analysis of the effects of spend on childhood obesity that took account of individual (pupil) characteristics as well as LA factors. Although pupil-level NCMP datasets exist, they have been heavily redacted to protect anonymity. The datasets are effectively unusable for an investigation of LA impacts. For example, in 2015/16 around 90 000 records of healthy weight or underweight children were stripped of their LA codes to protect pupil anonymity. Instead of removing the geographic identifiers, anonymity could be protected by reporting combined healthy weight and underweight categories: this would enable the effects of LA spending on outcomes to be tested more robustly.

Our finding on the spatial distribution and temporal persistence of childhood obesity supported findings from previous UK studies that the prevalence of obesity was higher in more deprived areas.^7^^,^^30^ Due to neighbourhood variations, interventions on childhood obesity need to be tailored to the specific needs of each local area by focussing on the most important obesogenic factors.^30^ Others argue that obesity is a complex multi-causal problem that requires more than simple interventions, particularly when these rely on individual motivation.^31^

Our own research, as well as evidence from the Local Government Association, showed that LAs were using a range of diverse approaches to tackle childhood obesity.^32^^,^^33^ However, qualitative work undertaken as a separate part of our study found scepticism about the effectiveness of local initiatives in the absence of coherent national strategies.^32^

In 2016, the Government published its national strategy, *Childhood Obesity: A Plan for Action,*^2^ acknowledging the complex and multifactorial nature of the problem and the consequent need for active engagement from schools, communities, families and individuals. The strategy included a raft of measures: one was a levy on the soft drinks industry, with revenue to be invested in encouraging physical activity and balanced diets in school children. However, the remaining measures were voluntary targets, pledges or ambitions. The strategy was criticised as ‘weak’ for failing to provide national strategies on advertising or promotion of junk-food, and legislation or regulation to support voluntary targets on sugar, fat and salt.^34^ While the move of public health to local government has enabled a wider range of interventions and policy considerations to be taken into account, there remain limits to what LAs can achieve alone.

## Supplementary Material

cky252_Supplementary_Figure
