# Supplementary figures and images for: Effects of local authority expenditure on childhood obesity
Source: Eur J Public Health. 2018 Dec 7;29(4):785–90. doi: 10.1093/eurpub/cky252 (PMC6660108; doi:10.1093/eurpub/cky252)

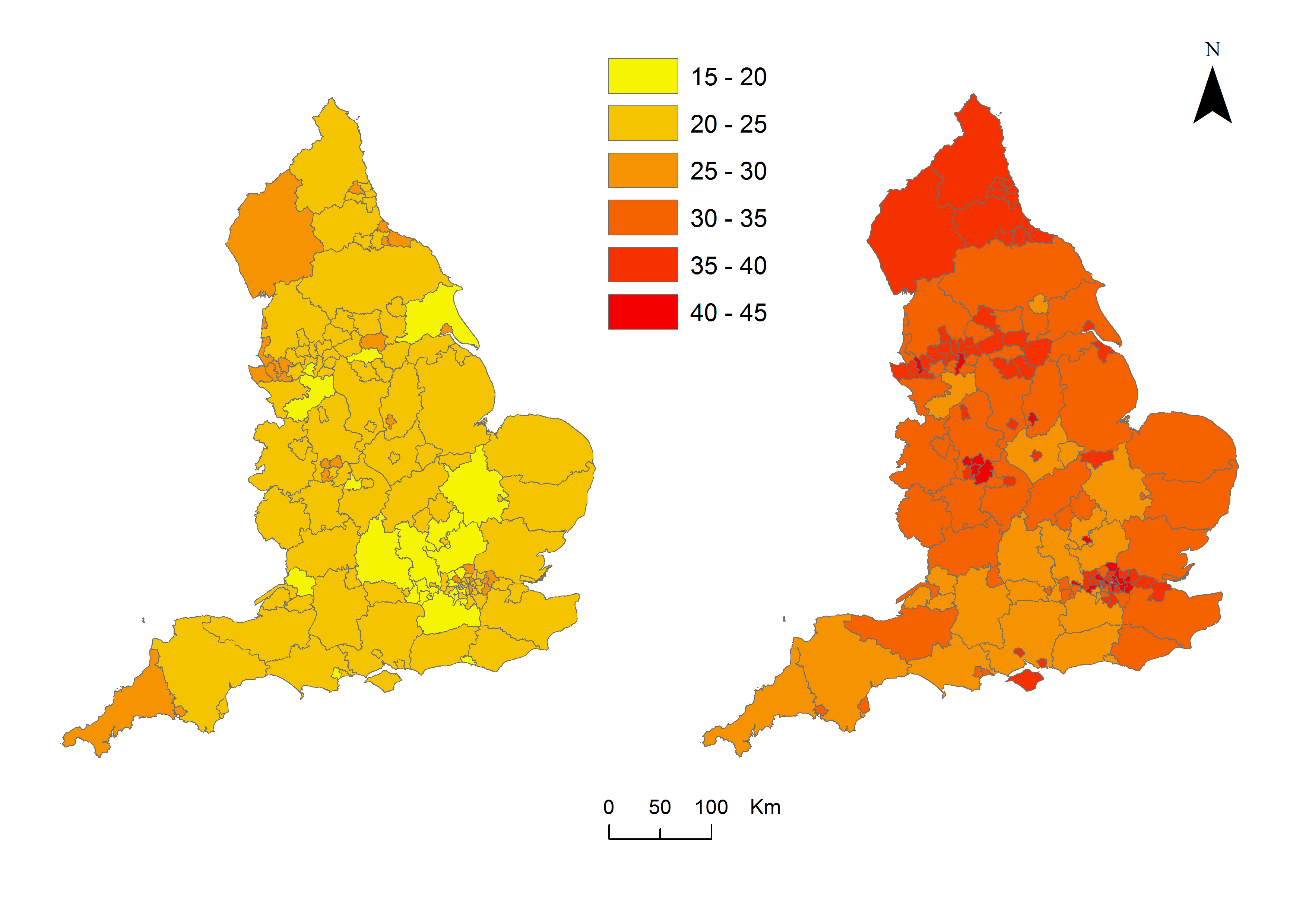

Supplement: cky252_Supplementary_Figure [file cky252_supplementary_figure.png]
